# Design and Reliability Analysis of a Novel Redundancy Topology Architecture

**DOI:** 10.3390/s22072582

**Published:** 2022-03-28

**Authors:** Fei Li, Wenyi Liu, Wanjia Gao, Yanfang Liu, Yanjun Hu

**Affiliations:** Key Laboratory of Instrumentation Science & Dynamic Measurement, Ministry of Education, North University of China, Taiyuan 030051, China; b1906056@st.nuc.edu.cn (F.L.); b1806014@st.nuc.edu.cn (W.G.); s2006067@st.nuc.edu.cn (Y.L.); 18234125986@163.com (Y.H.)

**Keywords:** redundancy, reliability, structural robustness

## Abstract

Topology architecture has a decisive influence on network reliability. In this paper, we design a novel redundancy topology and analyze the structural robustness, the number of redundant paths between two terminal nodes, and the reliability of the proposed topology by using natural connectivity and time-independent and time-dependent terminal pair reliability, k-terminal reliability, and all-terminal reliability comprehensively and quantitatively, and we compare these measures of the proposed topology with AFDX in three scenarios. The evaluations show that in the structural robustness analysis, when no nodes are removed, the natural connectivity of the proposed topology with 10 nodes, 16 nodes, and 20 nodes is 77.8%, 26.95%, and 81.39% higher than that of AFDX, respectively. In the time-independent reliability analysis, when the link reliability is 0.9, terminal pair reliability of the proposed topology with 10 nodes, 16 nodes, and 20 nodes is 5.78%, 17.75%, and 34.65% higher than that of AFDX, respectively; k-terminal reliability is 10.04%, 31.97%, and 53.74% higher than that of AFDX, respectively; and all-terminal reliability is 29.36%, 74.37%, and 107.91% higher than that of AFDX, respectively. In the time-dependent reliability analysis, when the operating time is 8000 h, the terminal pair reliability of the proposed topology with 10 nodes, 16 nodes, and 20 nodes is 3.53%, 10.87%, and 21.08% higher than that of AFDX, respectively; the k-terminal reliability is 6.20%, 19.65%, and 32.58% higher than that of AFDX, respectively; and the all-terminal reliability is 18.25%, 45.04%, and 63.86% higher than that of AFDX, respectively. The proposed topology increases the redundant paths of data transmission. It ensures reliable data transmission and has high robustness and reliability. It provides a new idea for improving the reliability of industrial buses.

## 1. Introduction

The dependability of buses is a matter gaining attention as the complexity of aerospace electronic systems and the amount of data transferred are rapidly increasing [1,2].

An important aspect of industrial buses is the underlying network topology architecture. The topology architecture has a decisive influence on the data delay, throughput, network area, and routing strategy of communication between nodes. Moreover, it is an important guarantee for reliable data transmission [3]. Network topology architecture is the geometric ordering of communication links and nodes in a network, which is used to represent the structural appearance of the entire network [4]. Common network topology architectures include bus topology [5], ring topology [6], star topology [7,8], and tree topology [9]. There are several papers comparing and evaluating these topology architectures. Astrit Ademaj et al. [10] evaluated bus topology architecture. Although it is simple in structure, convenient in construction, and low in cost, the main trunk link plays a decisive role in the entire network. The failure of the main trunk link will cause the entire network to be paralyzed. At the same time, there are single points of failure in the bus topology. As long as any node on the bus topology fails, the communication between the other nodes will be affected [8]. Philippe Ezran et al. [11] studied availability optimization in a ring-based topology network. The ring network has a simple structure and is easy to manage, but there are too many nodes, low transmission efficiency, and it is not easy to expand. Star topology is more robust than bus topology because it is less prone to common-mode failures. However, star topology increases the hardware cost and the probability of failures and errors, which have a negative impact on system reliability. Manuel Barranco et al. [12] quantitatively compared the error-containment capabilities of bus topology and star topology. Compared with bus topology, star topology improves the fault tolerance and error containment of the network. However, if the central node fails in the star topology, the entire network will be paralyzed. At the same time, the heavy burden of the central node is not conducive to expanding the utilization efficiency of the link. Abdelhafid, Bouhraoua et al. [13] used a tree topology. It is an extended form of the bus topology and is easy to expand and isolate failures. It is more extensible than the star topology, but it depends heavily on the root node.

Dependability of the system is the ability to deliver service that can justifiably be trusted [14]. Dependability is an integrative concept that encompasses the following attributes: reliability, availability, safety, security, performability, maintainability, and testability [14]. Among them, researchers pay the most attention to reliability evaluation [15,16]. In the process of data transmission, node or link failure happens from time to time, which leads to an inability to transmit the data reliably. Therefore, it is important to enhance the reliability of a system. One way to enhance the reliability of a system is to implement network redundancy. Network redundancy is classified into network device redundancy and network terminal redundancy. Network device redundancy is the redundancy of the switches and the links between the switches. When a single failure occurs on switches or the communication links between switches, the system can restore the communication by activating redundant switches or redundant links. Network terminal redundancy provides redundancy for all network components, including the links between nodes and switches. The terminal nodes are connected to two independent redundant subnets through two parallel ports. When a single failure occurs anywhere in the network, data can be transmitted through another normal subnet to achieve seamless switchover in the event of a failure. PRP protocol, a popular redundancy mechanism, is one of the network terminal redundancy protocols [17]. PRP protocol adopts a parallel network to transmit data. It has zero failure recovery time and can tolerate permanent failures. Moreover, it has the advantages of simple implementation, flexible topology, and no additional bandwidth [18], but it will increase the network cost and wiring complexity. In practice, secondary data allows a certain failure recovery time. If the PRP protocol is used to transmit the same data, the bandwidth usage will increase, resulting in bandwidth waste, and the advantages of parallel network cannot be fully utilized. Researchers have studied network device redundancy for star topology and ring topology. Manuel Barranco et al. [19] proposed a replicated star topology, called ReCANcentrate, which includes two hubs similar to the CANcentrate’s one and has no severe points of failure. David Gessner et al. [20] designed a media redundancy management driver of the star topology. It can enhance error-containment and fault-tolerance mechanisms. For the ring topology, Mohsen Jahanshahi and Fathollah Bistouni [6] proposed two ring redundancy topology architectures to enhance the reliability of the network. Ahmed Amari and Ahlem Mifdaoui [21] proposed duplicated mono-ring and multiple-ring topologies, which increase the reliability of the network. As far as we know, no researcher has designed redundancy for the tree topology to improve network reliability. Therefore, this paper designs a novel redundancy topology architecture based on tree topology.

Most of the literature deals with the correctness or real-time guarantee of the specific protocol mechanism during bus reliability analysis [22,23]. Zhong Zheng et al. [24] proposed a joint routing strategy and scheduling strategy that takes RC traffic into account. This allows both TT and RC traffic to be schedulable, and the worst-case end-to-end delay (WCD) of RC traffic can be reduced to meet the demand of the network. This will optimize the TTE network. Jun Lu et al. [25] presented a new architecture, SDTTE, to minimize the end-to-end delay, which can enhance real-time and determinacy of TT messages. Thus, these studies are not intended to assess how different topologies affect the capacity of nodes for communicating among themselves. For the evaluation of the bus, the first thing is to evaluate the structural robustness of the topology itself, and the second is how the reliability of the network changes when the nodes or links in the network are subjected to random failures. As far as we know, no researchers have conducted quantitative analysis on the structural robustness and reliability of aerospace electronic system buses. Therefore, this paper designs a novel, highly reliable redundancy topology architecture based on tree topology. We quantitatively evaluate the invulnerability and the survivability of the topology architecture and analyze the fault tolerance capability of the system. The topology architecture proposed in this paper provides a new idea for the reliability design of industrial buses.

Hence, in this paper, our main contributions are as follows:

- To meet the reliability requirements of the industrial buses, we propose a novel redundancy topology architecture. The proposed novel topology architecture ensures the reliable transmission of data in the network.
- The structural robustness and reliability of the proposed topology are analyzed by using natural connectivity, time-independent and time-dependent terminal pair reliability, k-terminal reliability, and all-terminal reliability comprehensively and quantitatively. In addition, these measures of the proposed topology are compared with AFDX in three scenarios.
- The proposed topology increases the redundant paths of data transmission. It ensures reliable data transmission and has high robustness and reliability. It provides a new idea for improving the reliability of industrial buses.

The paper is organized as follows. Related work is reviewed in Section 2. The proposed novel redundancy topology architecture is presented in Section 3. In Section 4, we quantitatively analyze the structural robustness of the proposed topology and compare it with the AFDX topology architecture. Section 5 presents the reliability analysis and quantitative reliability comparison of the proposed novel topology architecture with AFDX topology architecture. Finally, Section 6 concludes the paper.

## 2. Related Work

Recently, analysis of bus reliability has mainly focused on evaluating the performance of the specific protocol mechanism and the quality of service (QoS) of the network. Valerio Rosset et al. [22] presented the reliability models of a group membership protocol (GMP) and evaluated the reliability of the assumptions made in the proof of a GMP. The results showed that they are computationally practical for realistic configurations. Rodrigo Lange et al. [23] used an on-line scheduling approach to ensure real-time delivery of messages in the FlexRay static segment. Luxi Zhao et al. [26] determined the worst-case end-to-end delay of rate-constrained (RC) traffic in TTEthernet using network calculus, which reduces the pessimism of the RC analysis. Yuanyuan Xu et al. [27] proposed a scheduling method of an AFDX terminal system based on time-triggered and event-triggered, which ensures the different delay requirements of different data types of AFDX. David Gessner et al. [28] designed an FTT replicated star for Ethernet (FTTRS), a communication subsystem that tolerates permanent and transient faults, even if they occur simultaneously. It can guarantee hard, real-time requirements and increase the reliability of the network. Xueqian Tang et al. [29] performed the worst-case delay analysis using a revised trajectory approach for an AFDX network. Manuel Barranco et al. [30] quantitatively evaluated the mechanism to deal with faults of FTTRS [28]. Inés Álvarez et al. [31] proposed to use time redundancy to tolerate temporary faults in the links for time-sensitive networking (TSN), and evaluated the end-to-end delay, the jitter, and the bandwidth consumption of the network. The above-mentioned improvements of network reliability are all from the perspective of network protocols, and do not pay attention to the most basic factor affecting network reliability: topology architecture. To evaluate network reliability, the first thing is to evaluate the structural robustness and the survivability of the topology architecture. However, none of the above studies have evaluated network reliability from this perspective. There are also some studies on the optimization of network topology. Lei Zhang et al. [32] presented a multi-objective variable neighbourhood search algorithm (MOVNS) to optimize topology design. It guarantees the network performance of industrial Ethernet networks. Fanrong Kong et al. [33] proposed an algorithm to build a multi-objective model of switched industrial Ethernet topology structure to optimize topology design. However, they only proposed a method to optimize the topology of the network and did not design a novel topology architecture.

Table 1 summarizes the related work, research goals, and deficiencies. As can be seen, none of the existing research has focused on topology design. Although improving specific protocols can improve network reliability, this is based on a given topology architecture. Therefore, improving network reliability through a well-designed topology may be more effective than other methods. On the other hand, according to Table 1, another problem with previous works is the lack of accurate reliability analysis methods. Therefore, we designed a novel, highly reliable redundancy topology architecture and analyzed the invulnerability and the survivability of the network.

## 3. The Novel Redundancy Topology Architecture

AFDX and 1553B are commonly used in the control systems of aerospace electronic systems, but there are no more buses available for measurement systems. Our center has developed a novel bus for industrial measurement, which meets the requirements of large data transmission in measurement systems. It has the advantages of high speed, high real-time, high determinacy, and high reliability. In addition, it has been successfully applied in many aerospace projects.

**Figure 1:**
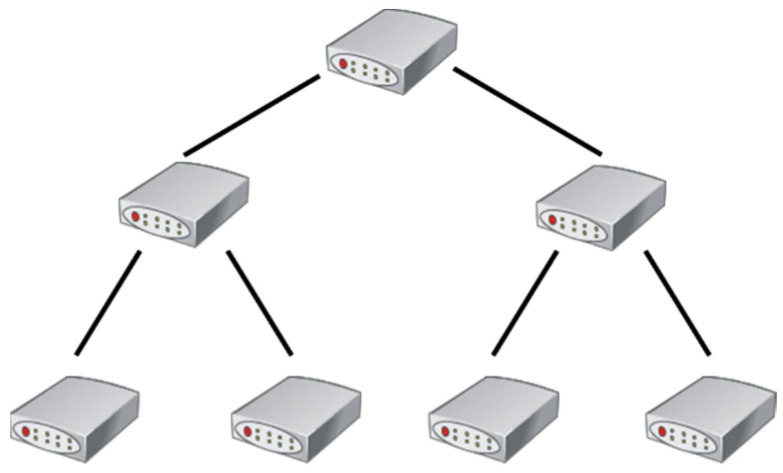
Schematic diagram of tree topology.

The network consists of switches and terminal devices interconnected through links. Topology architecture is the layout and connection form of switch nodes and link channels and is the foundation of the network. Tree topology is a tree-shaped arrangement of network nodes which can extend many branches and sub-branches. A schematic diagram of the tree topology is shown in Figure 1, where each node is a switch. The node at the top layer is the root node of the tree topology, and the remaining nodes are branch nodes. The nodes are connected by links. These nodes can forward data when terminal nodes communicate with each other.

**Figure 2:**
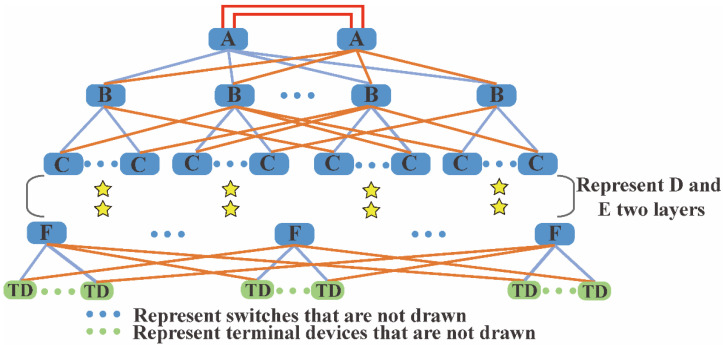
Schematic diagram of the novel redundancy topology architecture.

This paper proposes a novel redundancy topology architecture based on the tree topology. The schematic diagram is shown in Figure 2. The novel bus supports ring topology, tree topology, and mesh topology. For comparison, this paper chooses the mesh topology. The network supports switch cascading, with up to six layers. There are two switches on the top layer. Each switch has two uplink ports and a maximum of 16 downlink ports. The upper layer switch is connected to the lower layer switch through the downlink port. The terminal devices are located at the lowest layer and have two uplink ports similar to the switch. In Figure 2, the top layer switches are represented by the blue rectangle A. Similarly, switches located at layer two to layer six are represented by blue rectangle B, rectangle C, rectangle D, rectangle E, and rectangle F, respectively. The two top layer switches can be connected through two uplink ports to implement redundancy of the top layer switches, as shown by the two red lines in Figure 2. It can avoid a single point of failure of the top layer switches, which may paralyze the network. The two uplink ports of each switch located at layer two to layer six can be connected to two different switches of the upper layer to implement redundancy. They are shown by the blue lines and orange lines between the switches in Figure 2. In Figure 2, terminal devices are represented by green rectangle TD. Their two uplink ports can also be connected to two different switches of the upper layer to implement redundancy. They are shown by the blue lines and orange lines between the terminal devices and the switches in Figure 2. Redundant links between switches and terminal devices can improve the reliability of data transmission. We improved the reliability of the network by sacrificing the number of terminal devices that can be connected. The links are full-duplex, thus allowing communication in both directions, and the networks can be multi-hop. We can configure some or all ports as mesh redundant links for special applications, which can improve the reliability of the link connection, and can also balance and optimize the network transmission load.

## 4. Structural Robustness Analysis

To analyze the reliability of the network, it is necessary to analyze its structural robustness first. This is one of the most fundamental aspects of reliability analysis. The structural robustness of the network is also called the invulnerability of the network. It describes the impact of topology architecture on network reliability from the perspective of network connectivity. The indicators describing the structural robustness of the network include tenacity [34], integrity [35], adhesion [36], natural connectivity [37], etc. This paper selects natural connectivity to characterize the structural robustness of the network. Compared with other indicators, natural connectivity not only describes the difficulty of the network being destroyed, but also describes the severity of the network being destroyed. The most important thing is that it does not have the NP (non-deterministic polynomial) [38] problem that exists in the calculation of most indicators. Starting from the internal structural properties of the network, the natural connectivity describes the redundancy of alternative routes in the network by calculating the weighted sum of the number of closed walks of different lengths. Mathematically, natural connectivity is expressed as a special form of average eigenvalue. Equation (1) is used to calculate natural connectivity [37]. At the same time, natural connectivity changes monotonically when edges are added or deleted. This means that natural connectivity can accurately describe the nuances of the structural robustness of the network.

(1) $$\bar{\lambda }=\ln\left(\frac{1}{N}\sum_{i=1}^{N}e^{\lambda_{i}}\right)$$

where $\bar{\lambda }$ is natural connectivity, *N* is the total number of nodes in the network, and $\lambda_{i}$ is the eigenvalue of the adjacency matrix corresponding to the topology diagram.

**Figure 3:**
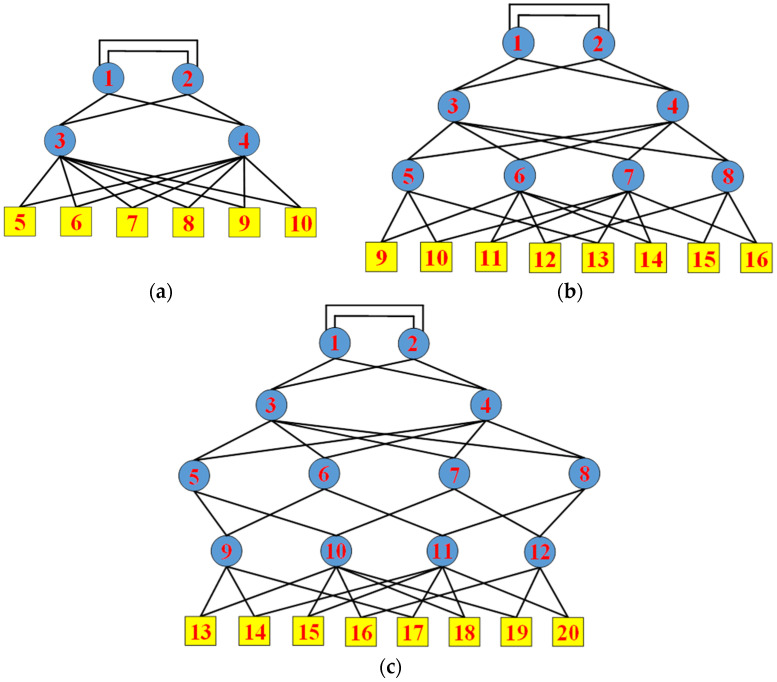
Redundancy topology diagram with (**a**) 10 nodes; (**b**) 16 nodes; and (**c**) 20 nodes.

To analyze the structural robustness of the novel redundancy topology proposed in this paper, we calculated the natural connectivity of the network in three scenarios. Three different networks with 10 nodes, 16 nodes, and 20 nodes, respectively, are shown in Figure 3. Node 1 to node 4 in Figure 3a, node 1 to node 8 in Figure 3b, and node 1 to node 12 in Figure 3c are switches, which are the backbone of the mesh redundancy topology. They transmit and process data exchanged between terminal devices. The top layer node 1 and node 2 are connected through two uplink links to implement the redundancy of the top layer switches. The two uplink ports of the remaining switches are connected to different switches at the upper layer for redundancy. Node 5 to node 10 in Figure 3a, node 9 to node 16 in Figure 3b, and node 13 to node 20 in Figure 3c are terminal devices. Data can be exchanged between any two terminal nodes. The two uplink ports of terminal devices are connected to different switches at the upper layer for redundancy. The black lines between the switches and between the switches and the terminal devices are links. There are 18 links in Figure 3a, 30 links in Figure 3b, and 38 links in Figure 3c, all of which are bidirectional links.

**Figure 4:**
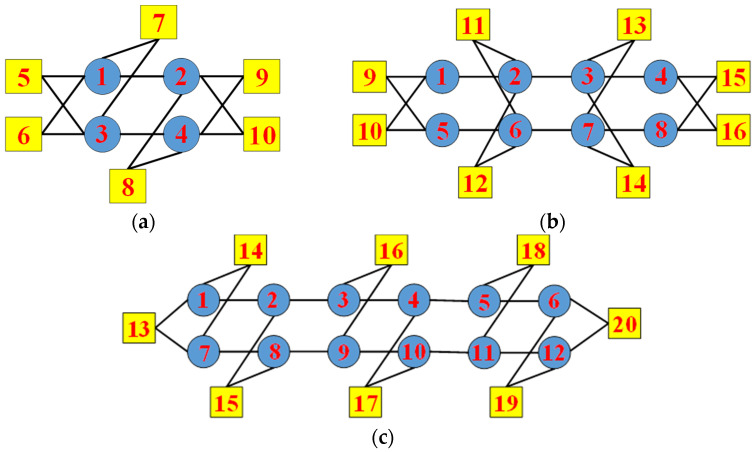
Topology diagram of AFDX with (**a**) 10 nodes; (**b**) 16 nodes; and (**c**) 20 nodes.

To verify the superiority of the novel topology architecture proposed in this paper, we compared it with AFDX, the popular fieldbus for aerospace electronic systems. AFDX adopts the star topology architecture. We chose to use the same number of switches and terminal nodes as in Figure 3, as shown in Figure 4. Node 1 to node 4 in Figure 4a, node 1 to node 8 in Figure 4b, and node 1 to node 12 in Figure 4c are switches, which are an important part of the AFDX star topology. Node 1 to node 2 and node 3 to node 4 in Figure 4a, node 1 to node 4 and node 5 to node 8 in Figure 4b, and node 1 to node 6 and node 7 to node 12 in Figure 4c are the two redundant sub-networks of AFDX, respectively. The switches in each sub-network can be cascaded. They transmit and process data exchanged between terminal devices. Node 5 to node 10 in Figure 4a, node 9 to node 16 in Figure 4b, and node 13 to node 20 in Figure 4c are terminal devices. Data can be exchanged between any two terminal nodes. The two ports of the terminal device are respectively connected to two redundant sub-networks to implement the redundant transmission of data. The black lines between the switches and between the switches and the terminal devices are links. There are 14 links in Figure 4a, 22 links in Figure 4b, and 26 links in Figure 4c, all of which are bidirectional links.

**Figure 5:**
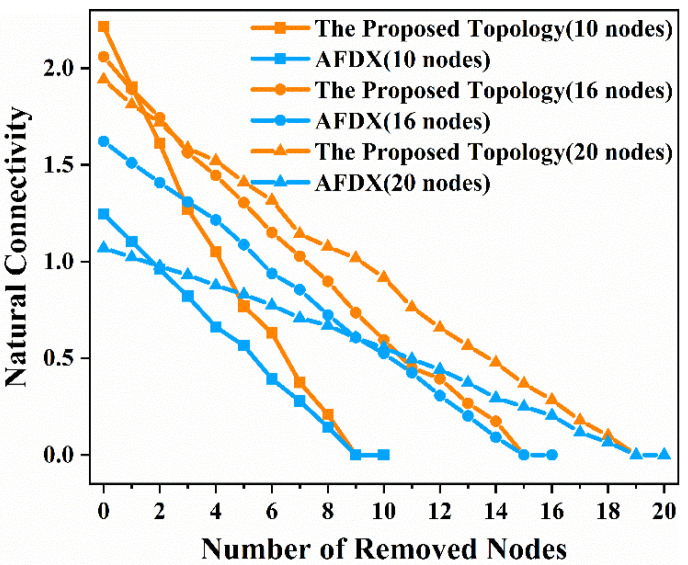
The comparison diagram of the natural connectivity of the proposed topology and AFDX with the number of removed nodes in the three scenarios.

When no nodes are removed from the network, the natural connectivity of the network can be calculated according to Equation (1). The greater the natural connectivity of the network, the higher the structural robustness of the network. In practice, some nodes in the network may fail, that is, the nodes may be disconnected from the network. Therefore, the number of nodes in the network will change, that is, the *N* in Equation (1) changes. At the same time, the adjacency matrix of the network will also change, so $\lambda_{i}$ in Equation (1) will change. Therefore, the natural connectivity of the network naturally changes. In this paper, we use the method of removing nodes randomly to simulate the random failure of nodes in the real network. We randomly removed 1 node to 10 nodes from Figure 3a and Figure 4a, 1 node to 16 nodes from Figure 3b and Figure 4b, and 1 node to 20 nodes from Figure 3c and Figure 4c, respectively, to analyze the changes in the natural connectivity of the network with the removed nodes. To make the results accurate, we repeated the experiment 100 times in each of the three scenarios and finally took the average value of the natural connectivity. The results of the natural connectivity of the network with 10 nodes, 16 nodes and 20 nodes, respectively, as a function of the number of removed nodes are shown in Figure 5.

As can be seen from the results in Figure 5, when there is no failure of nodes in the network, that is, when the total number of nodes in Figure 3a and Figure 4a is 10 nodes, the total number of nodes in Figure 3b and Figure 4b is 16 nodes, and the total number of nodes in Figure 3c and Figure 4c is 20 nodes, the natural connectivity of Figure 3a, Figure 4a, Figure 3b, Figure 4b, Figure 3c, and Figure 4c is 2.2144, 1.2455, 2.0592, 1.6221, 1.9412, and 1.0702, respectively. The natural connectivity of the proposed topology is 77.8%, 26.95%, and 81.39% higher than that of AFDX in the case of 10 nodes, 16 nodes, and 20 nodes, respectively. In other words, the proposed topology has higher structural robustness and is a more stable network than AFDX. When nodes in the network randomly fail, that is, when we randomly remove nodes from the network, and as the number of removed nodes increases, the natural connectivity of both the proposed topology and AFDX decreases in the three scenarios. However, it can be seen from Figure 5 that the natural connectivity of the proposed topology is always higher than that of AFDX in the three scenarios. Furthermore, using existing natural connectivity algorithm actually beautifies the communication links between nodes of AFDX in reality. Therefore, the structural robustness of the proposed topology is always higher than that of AFDX when nodes fail in the real network. Based on the above analysis of the natural connectivity of the proposed topology and AFDX, we can conclude that the proposed topology has higher structural robustness and is more robust and stable than AFDX.

## 5. Reliability Analysis

The invulnerability of the network is independent of the reliability of network components, so it cannot fully describe the reliability of the network. Therefore, in addition to analyzing the invulnerability of the network, we should also analyze the survivability of the network. Survivability is the characteristic of the network when it is subjected to random failures. We analyzed the survivability of the network, i.e., calculated the reliability of the network.

Reliability is defined by the IEEE as “the ability of a system or component to perform its required functions under stated conditions for a specified period of time” [39]. Reliability can be divided into time-independent reliability and time-dependent reliability. Time-independent reliability is the probability that the performance of the system meets its requirements under uncertain conditions when the system starts to run (t = 0). Time-dependent reliability is the probability that the system meets the requirements within the specified time within the life expectancy of the system (t > 0). In the reliability analysis of the network, there are three kinds of measures, namely terminal pair reliability, k-terminal reliability, and all-terminal reliability [16]. The terminal pair reliability of the network is defined as the probability that there is at least one fault-free path between the specified source node and the specified destination node. The definition of k-terminal reliability is the probability that the k nodes in a network can communicate with each other and there is a fault-free path. All-terminal reliability refers to the probability that there is a fault-free path between any node and other nodes in the network. In this paper, time-independent reliability and time-dependent reliability are calculated for these three kinds of measures and the reliability of the network is comprehensively analyzed.

**Figure 6:**
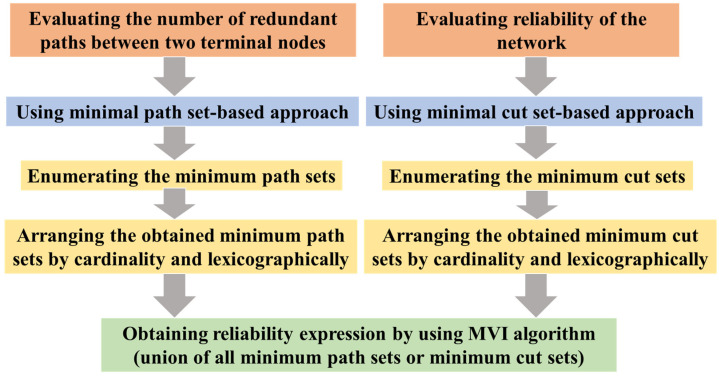
The flow chart of the overall evaluation process.

There are many methods to calculate reliability, such as the Monte Carlo method [40], continuous time Markov chains (CTMC) [41], and a reliability block diagram [42]. This paper analyzes the number of redundant paths between two terminal nodes using the minimal path set-based approach and the reliability of the network using the minimal cut set-based approach and hybrid multiple variable inversion (MVI) algorithm [43]. The flow chart of the overall evaluation process is shown in Figure 6. The first is to enumerate the minimum path sets or minimum cut sets of the network. Then we arrange the obtained minimum path sets or minimum cut sets by cardinality and lexicographically. Finally, the reliability expression is obtained according to the MVI algorithm. The reliability of the network is the probability of the union of all minimum path sets or all minimum cut sets between the communicating nodes in the network, in which the inclusion-exclusion principle is applied.

In a real network, link failure may occur due to various reasons such as aging, production flaws, and natural disasters such as earthquakes, and so on. Generally, a failure in a link occurs when the link is unable to pass the messages generated by nodes that are directly connected to the link. In addition, node failures necessarily induce link failures, and, moreover, introduce failures that are statistically dependent. Hence, the assumption of statistical independence requires the assumption of perfectly reliable nodes. In this paper, we focus on the reliability evaluation of the network topology, which should prevent the intervention of other factors on calculations (such as link failure rate). Therefore, in order to simplify theoretical and numerical calculations, it is necessary to assume that all of the links have identical reliability.

Therefore, in this paper, the following assumptions will be considered for the reliability analysis:

The nodes of the network are perfectly reliable, and only the failures of the links are considered.

The network links have only two states: working or failing.

Each link in the network may fail. The failure that occurs on a link is a permanent failure. After a link fails, it cannot be repaired.

All link failures are statistically independent.

All the links have identical reliability.

We analyzed the reliability of the network with 10 nodes, 16 nodes, and 20 nodes, as shown in Figure 3 and Figure 4, respectively. Section 5.1, Section 5.2 and Section 5.3 analyze terminal pair reliability, k-terminal reliability, and all-terminal reliability, respectively. Both time-independent and time-dependent reliability analyses are conducted for each measure.

### 5.1. Terminal Pair Reliability

In this section, we quantitatively analyzed the number of redundant paths when two terminal nodes communicate using the minimal path set-based approach. In addition, we calculated the time-independent and time-dependent terminal pair reliability of the network in the three scenarios.

To be reliable, the network must be fault tolerant. The basic idea of fault tolerance is to create redundant paths between any source node and destination node so that the redundant paths can be used when any link fails. The number of redundant paths can be used to analyze the fault tolerance capability of the network. Redundant paths of the network are all the possible paths between any source node and any destination node. We used the minimum path set-based method in [43] to generate all the possible minimum paths between two terminal nodes in the network. A minimal path can be defined as a sequence of links from source node to destination node without cycles. This method starts from the source node, finds the nodes connected to the source node, and, finally, finds the destination node, which is a path between the source node and the destination node, until all paths are found.

In this paper, we conducted the comparative analysis of the number of path sets, selecting node 5 and node 10 in Figure 3a and Figure 4a, node 9 and node 16 in Figure 3b and Figure 4b, and node 13 and node 20 in Figure 3c and Figure 4c, respectively. In the network with 10 nodes, there are 22 redundant paths between node 5 and node 10 in the proposed topology network compared with 2 redundant paths in AFDX. In the network with 16 nodes, there are 638 redundant paths between node 9 and node 16 in the proposed topology network compared with 2 redundant paths in AFDX. In the network with 20 nodes, there are 4182 redundant paths between node 13 and node 20 in the proposed topology network compared with 2 redundant paths in AFDX. It can be seen that as the number of nodes in the network increases, the gap of the number of redundant paths between the proposed topology network and AFDX becomes larger and larger. This indicates that when the network size is large, if one or more links in the network fail, the proposed topology network has more redundant paths that can be selected to continue data communication between terminal nodes. Therefore, the proposed topology provides more redundant paths and improves the fault tolerance capability of the system. The fault tolerance capability of the network enables the network to run continuously for a long time.

**Figure 7:**
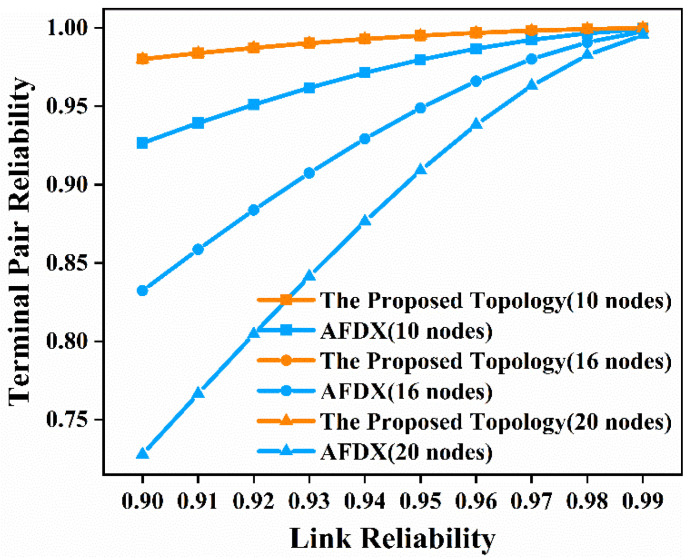
Time-independent terminal pair reliability comparison diagram of the proposed topology and AFDX in the three scenarios.

Next, we conducted the time-independent and time-dependent terminal pair reliability analysis, still selecting node 5 and node 10 in Figure 3a and Figure 4a, node 9 and node 16 in Figure 3b and Figure 4b, and node 13 and node 20 in Figure 3c and Figure 4c, respectively. The time-independent reliability of the network, that is, the reliability of the network, has nothing to do with time. When analyzing time-independent terminal pair reliability, we arranged the obtained minimal path sets by cardinality and lexicographically. Then, the time-independent terminal pair reliability expression was obtained using the MVI algorithm. What the MVI algorithm actually does is generate the union calculation of all minimal path sets. Meanwhile, we considered the influence of link reliability on time-independent terminal pair reliability. In this paper, we considered 10 cases where the link reliability was 0.9, 0.91, 0.92, 0.93, 0.94, 0.95, 0.96, 0.97, 0.98, and 0.99, respectively. The time-independent terminal pair reliability of the proposed topology network and AFDX with 10 nodes, 16 nodes, and 20 nodes, respectively, are shown in Figure 7.

It can be seen from the results in Figure 7 that when the link reliability is 0.9, the terminal pair reliability of the proposed topology network with 10 nodes is 0.9801 compared with 0.9266 for AFDX, the terminal pair reliability of the proposed topology network with 16 nodes is 0.9800 compared with 0.8323 for AFDX, and the terminal pair reliability for the proposed topology network with 20 nodes is 0.9800 compared with 0.7278 for AFDX. When the network has 10 nodes, 16 nodes, and 20 nodes, and the link reliability is 0.9, the terminal pair reliability of the proposed topology is 5.78%, 17.75%, and 34.65% higher than that of AFDX, respectively. It can be seen that as the number of nodes in the network increases, the terminal pair reliability of the proposed topology becomes larger and larger than that of AFDX. This shows that the proposed topology has incomparable advantages over AFDX when the network size is large in the communication between two terminal nodes. From Figure 7, we can see that as the link reliability increases, the terminal pair reliability of the proposed topology and AFDX both increase. Therefore, the improvement in link reliability leads to a significant improvement in the terminal pair reliability of the entire network. The terminal pair reliability of the network and AFDX approach each other, in the case of link reliability, and is 0.99. However, the proposed topology achieves undeniable advantages compared to AFDX in terms of terminal pair reliability. After the above analysis, we conclude that the proposed topology has higher terminal pair reliability than AFDX, especially in the case of lower link reliability. These results indicate the fact that using the proposed topology compared with AFDX leads to better performance even in undesirable conditions (lower link reliability). As the network size gradually becomes larger, this advantage becomes more and more obvious.

We have analyzed time-independent terminal pair reliability. In order to analyze network terminal pair reliability comprehensively, time-dependent terminal pair reliability should also be analyzed, as time is also an important aspect that affects the network reliability. The reliability of electronic components follows the bathtub curve over time [44]. The switches and terminal devices in the network are composed of many electronic components. Therefore, the reliability of the network will change over time. In this paper, we assume that the times-to-failure of the link is described with an exponential distribution [44]. Because this distribution is more suitable for modeling communication systems due to its memory-less property and accurate representation of electronic component time-to-failure, the reliability r of the link can be expressed as Equation (2) by the failure rate *λ* of the link.

(2) $$r=e^{-\lambda t}$$

where *r* is the reliability of the link, *λ* is the failure rate of the link, and *t* is time.

**Figure 8:**
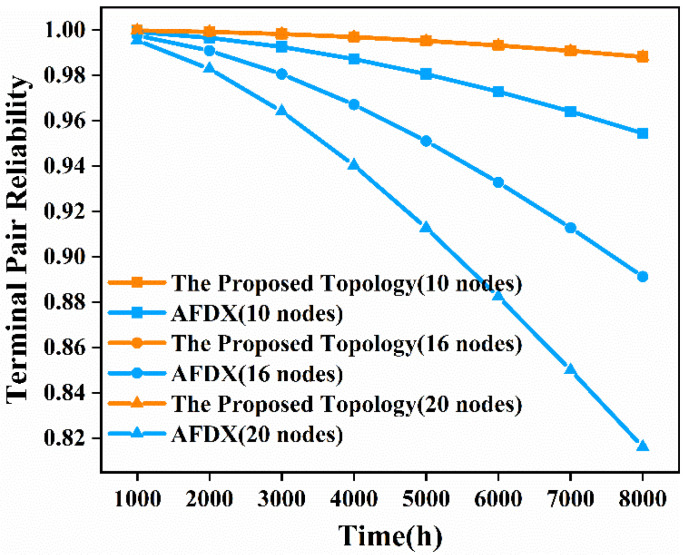
Time-dependent terminal pair reliability comparison diagram of the proposed topology and AFDX in the three scenarios.

We substituted Equation (2) into the already-obtained time-independent terminal pair reliability expression to obtain the time-dependent terminal pair reliability expression. This paper chooses *λ* as 10^−5^ per hour for comparison with AFDX. Therefore, the time-dependent terminal pair reliability is a function of time. We analyzed the time-dependent terminal pair reliability from 1000 h to 8000 h for the proposed topology network and AFDX with 10 nodes, 16 nodes, and 20 nodes, respectively. The results obtained are shown in Figure 8.

From Figure 8 we can see that as the operating time increases, the terminal pair reliability of the network decreases. When the operating time is 8000 h, the terminal pair reliability of the proposed topology network with 10 nodes, 16 nodes, and 20 nodes is 3.53%, 10.87%, and 21.08% higher than that of AFDX, respectively. Therefore, the proposed topology can run longer than AFDX in the case of the required reliability in the network when the two terminal nodes are communicating. As the number of nodes in the network increases, the terminal pair reliability of the proposed topology declines at basically the same rate, while AFDX declines more and more rapidly. This indicates that when the network size is large, the communication between the two terminal nodes of AFDX cannot achieve both in terms of runtime and reliability. However, both runtime and reliability can be guaranteed when the two terminal nodes of the proposed topology communicate with each other.

Through the above quantitative, comparative analysis of the time-independent and time-dependent terminal pair reliability between the proposed topology and AFDX in the three scenarios, we can conclude that the communication between the two terminal nodes of the proposed topology has obvious advantages over AFDX in both network size and runtime.

### 5.2. k-Terminal Reliability

In the previous section, we had analyzed the number of redundant paths between terminal nodes and time-independent and time-dependent terminal pair reliability of the network. In this section, we calculate the time-independent and time-dependent k-terminal reliability of the network in the three scenarios. Unlike terminal pair reliability, which considers the communication between two fixed terminal nodes in the network, k-terminal reliability considers the communication among k-terminal nodes in the network.

**Figure 9:**
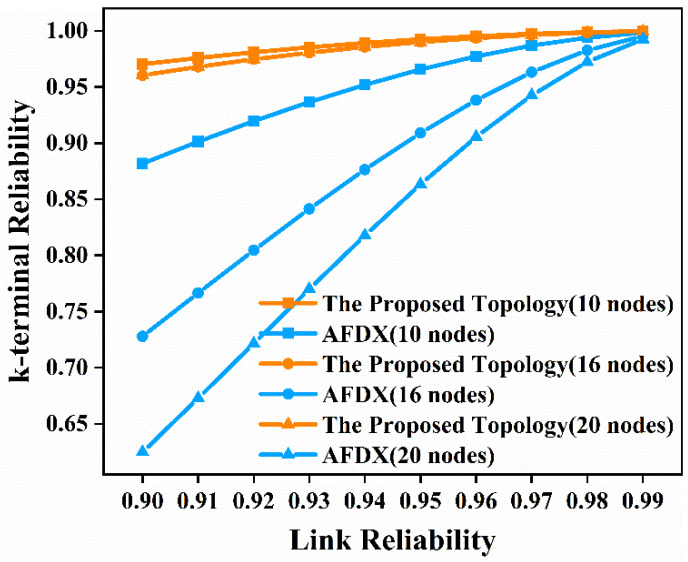
Time-independent, k-terminal reliability comparison diagram of the proposed topology and AFDX in the three scenarios.

In this paper, we respectively select node 5, node 7, and node 10 in Figure 3a and Figure 4a; node 9, node 12, node 14, and node 16 in Figure 3b and Figure 4b; and node 13, node 16, node 18, and node 20 in Figure 3c and Figure 4c to analyze the time-independent and time-dependent k-terminal reliability of the network. We first enumerate the minimum cut sets of the network using the minimum cut set-based approach. The minimum cut set of the network means that if the links in the minimum cut set are disconnected, the terminal nodes that need to communicate in the network will not be able to communicate with each other. If one or more of these links are removed, the terminal nodes can still communicate with each other, then it cannot be called the minimum cut set of the network. For example, the links connecting node 10 to node 3 and node 4 in Figure 3a are a minimal cut set of the network. We use this method to enumerate all the minimal cut sets of the network and arrange the obtained cut sets by cardinality and lexicographically. Then we use the MVI algorithm to obtain the time-independent, k-terminal reliability expression. What the MVI algorithm actually does is generate the union calculation of all minimal cut sets. Meanwhile, we still consider the influence of link reliability on time-independent, k-terminal reliability. In this paper, we still considered 10 cases where the link reliability is 0.9, 0.91, 0.92, 0.93, 0.94, 0.95, 0.96, 0.97, 0.98, and 0.99, respectively. The time-independent, k-terminal reliability of the proposed topology network and AFDX with 10 nodes, 16 nodes, and 20 nodes, respectively, are shown in Figure 9.

As can be seen from the results in Figure 9, when the link reliability is 0.9, the k-terminal reliability of the proposed topology network with 10 nodes is 0.9703 compared with 0.8817 for AFDX, the k-terminal reliability of the proposed topology network with 16 nodes is 0.9605 compared with 0.7278 for AFDX, and the k-terminal reliability for the proposed topology network with 20 nodes is 0.9605 compared with 0.6247 for AFDX. When the network has 10 nodes, 16 nodes, and 20 nodes, and the link reliability is 0.9, the k-terminal reliability of the proposed topology is 10.04%, 31.97%, and 53.74% higher than that of AFDX, respectively. It can be seen that as the number of nodes in the network increases, the percentage of improvement in k-terminal reliability of the proposed topology is greater and greater than AFDX. This shows that the proposed topology has obvious advantages over AFDX in terms of k-terminal nodes communicating with each other when the network size is large. From Figure 9, we can see that as the link reliability increases, the k-terminal reliability of the proposed topology and AFDX both increase. Although the k-terminal reliability of the two networks is very close when the link reliability is 0.99, the proposed topology has an undeniable advantage over AFDX when the link reliability is low. After the above analysis, we conclude that the proposed topology has higher k-terminal reliability than AFDX, especially in the case of lower link reliability. These results indicate the fact that using the proposed topology compared with AFDX leads to better performance, even in lower link reliability, when k-terminal nodes communicate with each other.

**Figure 10:**
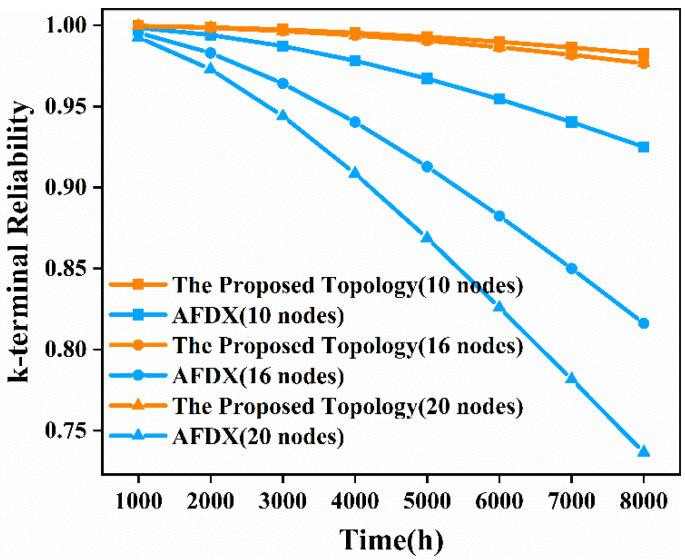
Time-dependent, k-terminal reliability comparison diagram of the proposed topology and AFDX in the three scenarios.

Similar to the analysis of the terminal pair reliability of the network, we also need to analyze the time-dependent, k-terminal reliability of the network. Similarly, we substitute Equation (2) into the already-obtained time-independent, k-terminal reliability expression to obtain the time-dependent, k-terminal reliability expression. We still choose *λ* as 10^−5^ per hour for comparison with AFDX. Then we analyze the time-dependent, k-terminal reliability from 1000 h to 8000 h for the proposed topology network and AFDX with 10 nodes, 16 nodes, and 20 nodes, respectively. The results obtained are shown in Figure 10.

As can be seen from Figure 10, as the operating time increases, the k-terminal reliability of the network decreases. When the operating time is 8000 h, the k-terminal reliability of the proposed topology network with 10 nodes, 16 nodes, and 20 nodes is 6.20%, 19.65%, and 32.58% higher than that of AFDX, respectively. Therefore, the k-terminal nodes of the proposed topology can communicate with each other for a longer time than AFDX under the required reliability. The k-terminal reliability of the proposed topology decreases significantly slower than AFDX as the network size increases and the runtime goes on. This indicates that when the network size is large and the runtime is long, the reliability of communication among k-terminal nodes in the proposed topology is superior to that of AFDX.

Through the above quantitative, comparative analysis of the time-independent and time-dependent, k-terminal reliability between the proposed topology and AFDX in the three scenarios, we can conclude that the reliability of k-terminal nodes communicating with each other in the proposed topology network is higher than that of AFDX, even in the case of large network size and long runtime.

### 5.3. All-Terminal Reliability

In practice, we need to consider the reliability of all the terminal nodes communicating with each other in the network. Therefore, it is important to analyze the all-terminal reliability of the network. Only by analyzing the all-terminal reliability of the network can we conduct the comprehensive reliability analysis of the network. All-terminal reliability of the network is the reliability of communication among all terminal nodes in the network. The terminal pair reliability and k-terminal reliability analyzed above are special cases of al- terminal reliability. Therefore, in this section, we calculated the time-independent and time-dependent, all-terminal reliability of the network in the three scenarios.

**Figure 11:**
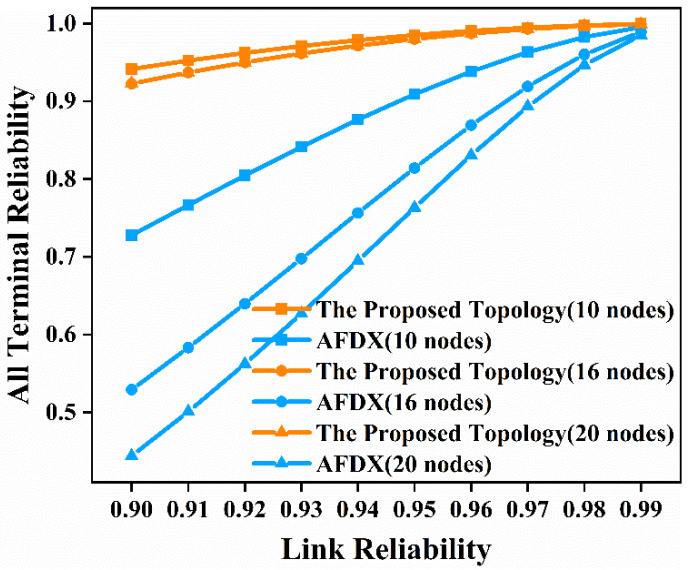
Time-independent, all-terminal reliability comparison diagram of the proposed topology and AFDX in the three scenarios.

In this paper, we analyze the reliability of all terminal nodes communicating with each other in the network. That is, we respectively select node 5 to node 10 in Figure 3a and Figure 4a, node 9 to node 16 in Figure 3b and Figure 4b, and node 13 to node 20 in Figure 3c and Figure 4c to analyze all-terminal reliability of the network. We use the minimum cut set-based method to enumerate all the minimal cut sets of the network and arrange the obtained cut sets by cardinality and lexicographically. Then we use the MVI algorithm to obtain the time-independent, all-terminal reliability expression. In this paper, we still considered 10 cases where the link reliability is 0.9, 0.91, 0.92, 0.93, 0.94, 0.95, 0.96, 0.97, 0.98, and 0.99, respectively, and analyzed their influence on the all-terminal reliability of the network. The time-independent, all-terminal reliability of the proposed topology network and AFDX with 10 nodes, 16 nodes, and 20 nodes, respectively, are shown in Figure 11.

As can be seen from the results in Figure 11, when the link reliability is 0.9, the all-terminal reliability of the proposed topology network with 10 nodes is 0.9415 compared with 0.7278 for AFDX, the all-terminal reliability of the proposed topology network with 16 nodes is 0.9226 compared with 0.5291 for AFDX, and the all-terminal reliability for the proposed topology network with 20 nodes is 0.9226 compared with 0.4438 for AFDX. When the network has 10 nodes, 16 nodes, and 20 nodes, and the link reliability is 0.9, the all-terminal reliability of the proposed topology is 29.36%, 74.37%, and 107.91% higher than that of AFDX, respectively. It can be seen that with the increase in number of nodes in the network, the percentage of improvement in the all-terminal reliability of the proposed topology is increasingly greater than AFDX. This shows that the proposed topology has obvious advantages over AFDX in terms of all terminal nodes communicating with each other when the network size is large. From Figure 11, we can see that as the link reliability increases, the all-terminal reliability of the proposed topology and AFDX both increase. Although the all-terminal reliability of the proposed topology and AFDX is very close when the link reliability is 0.99, the proposed topology has an undeniable advantage over AFDX when the link reliability is low. After the above analysis, we conclude that the proposed topology has higher all-terminal reliability than AFDX, especially in the case of lower link reliability. These results show that compared to AFDX, there is better performance with the proposed topology when all terminal nodes communicate with each other, even in lower link reliability.

**Figure 12:**
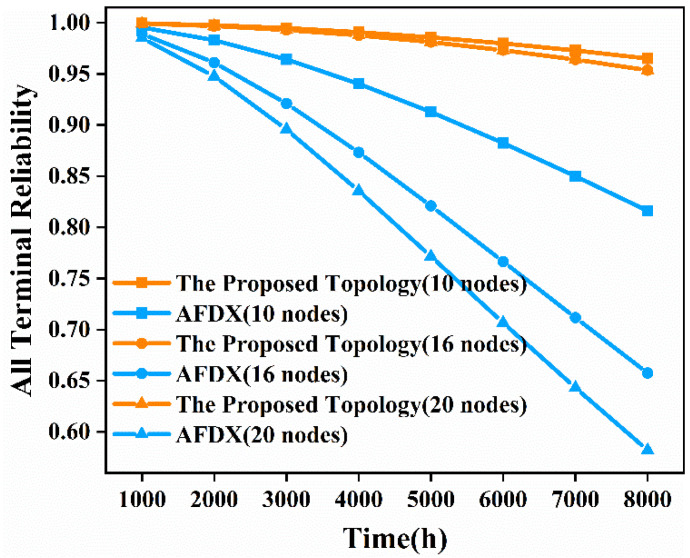
Time-dependent, all-terminal reliability comparison diagram of the proposed topology and AFDX in the three scenarios.

Next, we analyze the time-dependent, all-terminal reliability of the network similarly. We still substitute Equation (2) into the already-obtained time-independent, all-terminal reliability expression to obtain the time-dependent, all-terminal reliability expression. We still choose *λ* as 10^−5^ per hour for comparison with AFDX. Then we analyze the time-dependent, all-terminal reliability from 1000 h to 8000 h for the proposed topology network and AFDX with 10 nodes, 16 nodes, and 20 nodes, respectively. The results obtained are shown in Figure 12.

As can be seen from Figure 12, as the operating time increases, the all-terminal reliability of the network decreases. When the operating time is 8000 h, the all-terminal reliability of the proposed topology network with 10 nodes, 16 nodes, and 20 nodes is 18.25%, 45.04%, and 63.86% higher than that of AFDX, respectively. Therefore, the all-terminal nodes of the proposed topology can communicate with each other for a longer time than AFDX under the required reliability. The all-terminal reliability of the proposed topology decreases significantly slower than AFDX as the network size increases and the runtime goes on. This indicates that when the network size is large and the runtime is long, the reliability of communication among all terminal nodes in the proposed topology is superior to that of AFDX.

According to the above quantitative analysis of the number of redundant paths between two terminal nodes and the time-independent and time-dependent terminal pair reliability, k-terminal reliability, and all-terminal reliability of the network, we conclude that the proposed topology has undeniable advantages over AFDX in these three measures, no matter in the case of large number of nodes in the network or low link reliability.

## 6. Conclusions

Based on the tree topology, we designed a reliable, novel, redundancy topology architecture. Compared with the traditional topology architecture, this topology increases redundant paths to ensure reliable data transmission. First, we described the novel redundancy topology architecture. Then, we chose the novel redundancy topology with 10 nodes, 16 nodes, and 20 nodes, respectively, to analyze structural robustness using natural connectivity. We studied the change of natural connectivity with removed nodes. Next, we quantitatively analyzed the number of redundant paths between two terminal nodes and survivability using time-independent and time-dependent terminal pair reliability, k-terminal reliability, and all-terminal reliability. When analyzing time-independent reliability, we studied the influence of 10 different link reliability scenarios on network reliability. When analyzing time-dependent reliability, we studied the influence of runtime on network reliability. Meanwhile, we quantitatively compared the structural robustness and reliability of the proposed topology with AFDX. The experimental results show that in the structural robustness analysis, when no nodes are removed from the network, the natural connectivity of the proposed topology with 10 nodes, 16 nodes, and 20 nodes is 77.8%, 26.95%, and 81.39% higher than that of AFDX, respectively. When the nodes are removed, the natural connectivity of the proposed topology is always greater than that of AFDX in the three scenarios. In the time-independent reliability analysis, when the link reliability is 0.9, the terminal pair reliability of the proposed topology with 10 nodes, 16 nodes, and 20 nodes is 5.78%, 17.75%, and 34.65% higher than that of AFDX, respectively; the k-terminal reliability is 10.04%, 31.97%, and 53.74% higher than that of AFDX, respectively; and the all-terminal reliability is 29.36%, 74.37%, and 107.91% higher than that of AFDX, respectively. As the link reliability increases, the three reliability measures of the proposed topology and AFDX increase, but the reliability of the proposed topology is always higher than that of AFDX. In the time-dependent reliability analysis, when the operating time is 8000 h, the terminal pair reliability of the proposed topology with 10 nodes, 16 nodes, and 20 nodes is 3.53%, 10.87%, and 21.08% higher than that of AFDX, respectively; the k-terminal reliability is 6.20%, 19.65%, and 32.58% higher than that of AFDX, respectively; and the all-terminal reliability is 18.25%, 45.04%, and 63.86% higher than that of AFDX, respectively. The evaluations show that the structural robustness of the proposed topology is higher than that of AFDX and the network is more robust and stable. The proposed topology increases the redundant paths of data transmission and increases time-independent and time-dependent terminal pair reliability, k-terminal reliability, and all-terminal reliability of the network. It guarantees the reliable transmission of data and improves the reliability of the network. This paper conducts a comprehensive, quantitative analysis of the structural robustness and reliability of the proposed topology and proves the reliability of the proposed topology architecture. The reliable, novel, redundancy topology architecture designed in this paper provides an effective solution for the reliability design of industrial buses.

## Figures and Tables

**Table 1:** Related works and their deficiencies.

Related Work	Goal	Deficiencies
[22]	Presenting the reliability models for reliability evaluation	The novel topology is not designed. The reliability of the topology itself is not evaluated.
[23]	Using an on-line scheduling approach to guarantee real-time transmission of messages	The novel topology is not designed. The reliability of the topology itself is not evaluated.
[26]	Performing the end-to-end delay analysis of RC traffic	The novel topology is not designed. The reliability of the topology itself is not evaluated.
[27]	Proposing a scheduling method to guarantee the delay requirements	The novel topology is not designed. The reliability of the topology itself is not evaluated.
[28]	Proposing FTTRS for Ethernet to ensure hard, real-time requirements	The novel topology is not designed. The reliability of the topology itself is not evaluated.
[29]	Performing the end-to-end delay analysis for AFDX	The novel topology is not designed. The reliability of the topology itself is not evaluated.
[30]	Quantitatively evaluating FTTRS [28]	The novel topology is not designed. The reliability of the topology itself is not evaluated.
[31]	Using time redundancy to tolerate temporary faults	The novel topology is not designed. The reliability of the topology itself is not evaluated.
[32]	Proposing an algorithm to optimize topology design	The novel topology is not designed. The reliability of the topology itself is not evaluated.
[33]	Proposing an algorithm to optimize topology design	The novel topology is not designed. The reliability of the topology itself is not evaluated.
